# Osteomyelitis with abscess associated with acute closed upper humerus fracture in an adult: A case report

**DOI:** 10.1002/ccr3.7640

**Published:** 2023-07-10

**Authors:** John G. Skedros, Tyler R. Smith, John T. Cronin

**Affiliations:** ^1^ Utah Orthopaedic Specialists Salt Lake City Utah USA; ^2^ Saint Marks Medical Center Salt Lake City Utah USA; ^3^ University of Utah School of Medicine Salt Lake City Utah USA

**Keywords:** abscess, acute osteomyelitis, fracture nonunion, infection, proximal humerus fracture

## Abstract

A 64‐year‐old female presented with malaise and fever 11 days after a closed, minimally displaced humerus surgical neck fracture. MR imaging revealed an abscess around the fracture, which is a very rare occurrence in adults. Two open debridements and IV antibiotics eradicated the infection. Reverse total shoulder arthroplasty was eventually performed for fracture nonunion.

## Graphical abstract

Coronal MR image of the right shoulder showing abscess formation within and around a closed fracture of the proximal humerus.

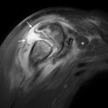

## 1 INTRODUCTION

Deep infection at the site of an acute closed fracture is very rare in an adult. In fact, we were able to identify just 15 adult cases of osteomyelitis occurring acutely at the site of any closed appendicular bone fracture since the late 1950s (Table 1).^1^, ^2^, ^3^, ^4^, ^5^, ^6^, ^7^, ^8^, ^9^, ^10^, ^11^ Of the 15 cases, only five were at the site of proximal humerus, similar to our patient.^3^, ^5^, ^6^, ^7^, ^8^ Immunosuppression appears to be a major risk factor in these cases.^12^, ^13^

**TABLE 1:** Acute infections of closed fractures (appendicular bones) in adults.

Reference	Patient age, sex	Fracture/infection site	Time from injury	Possible primary infection source	Organism	Factors affecting infection resistance	Antibiotic treatment^a^	Outcome
Cozen 1958^a^	Adult, F	Proximal tibia	5.5 months	NA	*Staphylococcus* sp.	NA	NA	Nonunion
	60, M	Femoral neck	5 months	NA	*Pseudomonas* sp.	Rheumatoid arthritis	Polymyxin	Femoral head resection
Watson and Whitesides 1976	58, F	Humeral neck	4 days	NA	Penicillin‐resistant *Staphylococcus aureus*	Chronic alcoholism	Nafcillin	Likely humeral head resection
	61, F	Humeral neck	20 days	NA	Group B beta‐hemolytic *Streptococcus*	Diabetes	Penicillin	Likely humeral head resection
	96, F	Femoral neck	12 days	NA	*Klebsiella pneumoniae*	NA	Gentamicin, penicillin	Femoral head resection
	59, M	Humeral neck	22 days	NA	*Bacteroides*	Diabetes	Primarily polymixcin and kanamycin; chloramphenicol, tetracycline and nitrofurantoin	Died
Aluisio and Scully 1996	61, F	Humeral neck	6 weeks	NA	*Salmonella enteriditis* & Methicillin‐resistant *Staphylococcus aureus*	Diabetes	Ciprofloxacin, vancomycin	Humeral head and shaft resection (8 cm)
Baskaran et al. 2004	38, F	Femoral diaphysis	15 days	NA	Methicillin‐sensitive *Staphylococcus aureus*	NA	Gentamicin‐impregnated beads, cloxacillin	2 cm shorter limb
Martínez et al. 2006	88, M	Humeral neck	8 days	Indwelling catheter	*Escherichia coli*	Bladder carcinoma	Amoxicillin/clavulanate, ciprofloxacin, clindamycin, cotrimoxazole	Died
Weidle et al. 2009^a^	77, F	Distal radius	3 days	Pneumonia	Group A beta‐hemolytic *Streptococcus*	Diabetes, bronchial carcinoma	Linezolid, meropenem, clindamycin	Died, necrotizing fasciitis
Kim and Tufesco 2012	31, F	Patella	2 days	NA	Group A beta‐hemolytic *Streptococcus*	NA	Cefazolin, ceftriaxone, cefalexin	No long‐term deficits
Baruah et al. 2016^a^	50, M	Humeral diaphysis	14 days	NA	*Staphylococcus aureus*	NA	Cephalosporin	Fibular graft
Kocutar et al. 2016	54, M	Clavicle	24 days	Toe wound	*Escherichia coli*	Chronic alcoholism, smoker	Floxacillin, cefotaxime	Died
Gellman et al. 2018	28, M	Humeral diaphysis	11 days	Chest tube, skin abrasions	Methicillin‐sensitive *Staphylococcus aureus*	NA	Cefazolin, gentamicin‐impregnated beads, cloxacillin, rifampicin	No long‐term deficits
Current case 2022	64, F	Humeral neck	13 days	NA	Methicillin‐sensitive *Staphylococcus aureus*	Chronic alcoholism	Cefazolin, vancomycin impregnated absorbable calcium sulfate beads	Reverse total shoulder arthroplasty

Abbreviations: F, female; humeral neck, surgical neck of the humerus; M, male; NA, not available/not known.

^**a**^ Dosage and duration of antibiotics not specified in original study.

**FIGURE 1:**
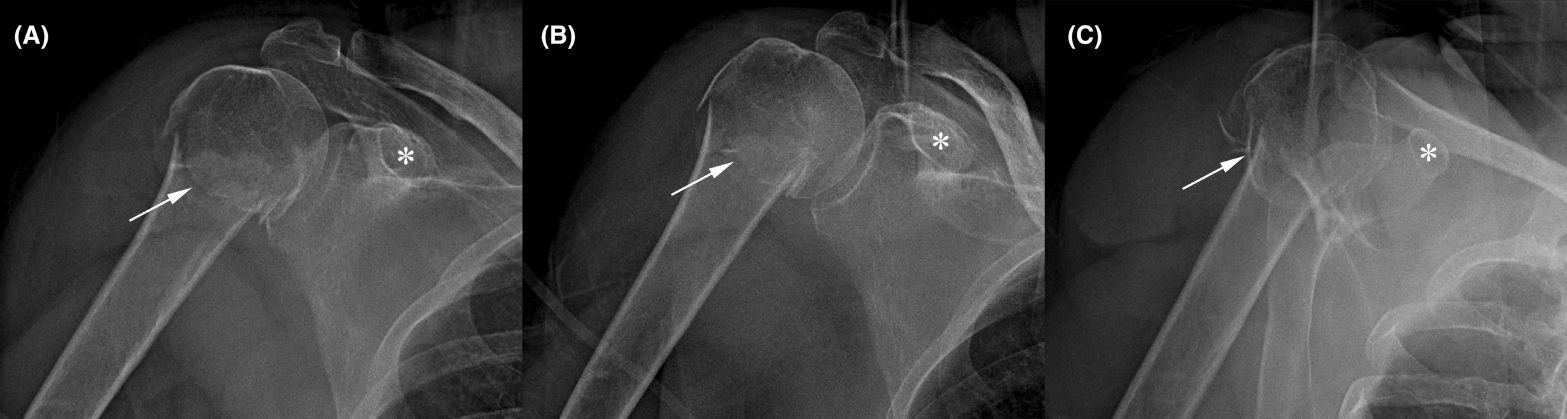
Radiographs showing our patient's minimally displaced and mildly comminuted two‐part surgical neck humerus fracture in anterior–posterior (A, B) and lateral (C) views. Asterisks (*) indicate the coracoid process and arrows indicate the fracture. Mild lucency along the fracture site seems consistent with fracture of poor‐quality bone, but some readers might consider this as subtle lysis to be consistent with a subacute infection.

We present the case of a 64‐year‐old female with chronic alcoholism who had osteomyelitis at the site of a closed minimally displaced surgical neck humerus fracture that resulted from a ground‐level fall (Figure 1). The infection was eradicated with two open surgical debridements and 6 weeks of intravenous (IV) antibiotics for the methicillin‐sensitive *Staphylococcus aureus* (MSSA) that grew in tissue cultures. At the second debridement, absorbable calcium sulfate beads loaded with tobramycin and vancomycin were implanted within and around the fracture site, but the fracture did not heal. A reverse total arthroplasty was ultimately done to restore shoulder function.

## 2 CASE PRESENTATION

A 64‐year‐old female with chronic alcoholism was admitted to a local hospital on September 6, 2020, after falling while intoxicated earlier that same day. Radiographs revealed a minimally displaced and mildly comminuted surgical neck fracture of the right humerus (Figure 1). Although examination of the radiographs revealed reduced bone density consistent with chronic alcoholism,^14^ the admitting physicians and a radiologist concluded that the relatively obvious lytic changes that are typically seen in chronic osteomyelitis were not present.^15^, ^16^ After a 3‐day hospitalization that focused on correcting hyponatremia, she was discharged with the anticipation of nonoperative treatment.

At the initial hospitalization, white blood cell count was normal and there was no evidence of a urinary tract infection or other known or suspected location of a chronic infection. Inflammatory markers were not obtained. She was also afebrile and denied any prior fevers/chills/sweats or right shoulder or upper arm swelling, pain, or dysfunction.

She returned to the same hospital 7 days later with malaise, tactile fevers, chills, and increasing pain around the fracture site. Her vital signs included blood pressure of 104/71, oral temperature 36.1°C, pulse 78 beats/minute, respiratory rate 16/minute, and oxygen saturation at 97% (finger oximeter). Leukocytosis (28,000/mm^3^) and high C‐reactive protein at 36.1 (normal is <1 mg/dL) raised concern for emerging sepsis, prompting transfer to our tertiary medical center. Erythrocyte sedimentation rate (ESR) was normal at 18 (normal range 0–30 mm/hr). Blood cultures were obtained and a fluoroscopic aspiration of her right shoulder joint revealed mildly turbid non‐serosanguinous fluid. IV antibiotic (Zosyn®, piperacillin and tazobactam) treatment was started.

On the third hospital day at our tertiary hospital, MSSA grew from the blood and shoulder fluid samples, and an orthopedic consultation was requested. Arthroscopic debridement was then promptly done by JGS and revealed minimal evidence of infection (three milliliters of mildly turbid non‐serosanguinous fluid and mild glenohumeral synovitis). Additional cultures were not obtained from that debridement. A drain was placed in the glenohumeral joint and IV antibiotic (Zosyn®, piperacillin and tazobactam) treatment continued.

**FIGURE 2:**
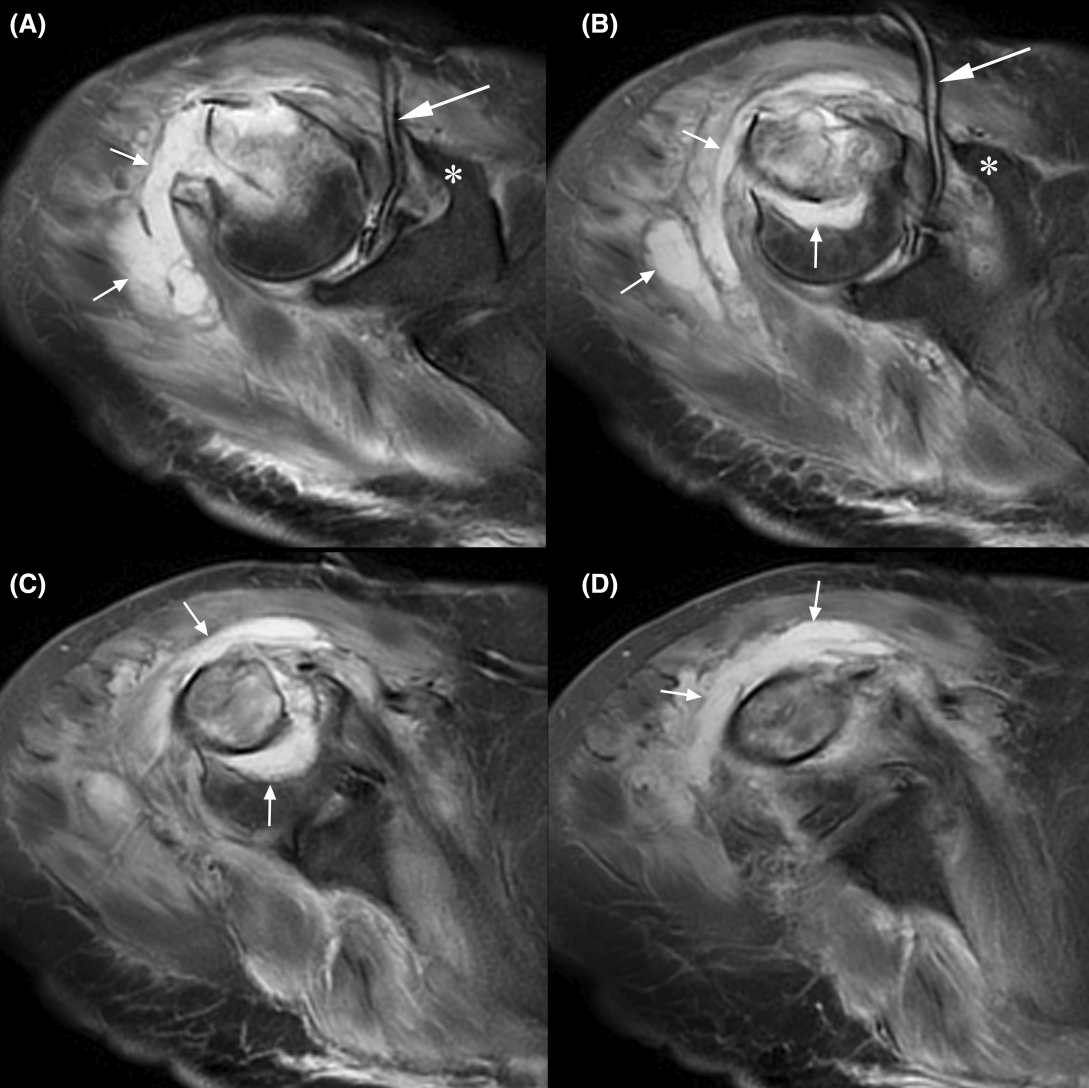
Sequential T2 (fluid enhancing) MR images in coronal view (A is most proximal, D is most distal). Asterisks (*) indicate the coracoid process, longer arrows indicate a Jackson–Pratt drain within the glenohumeral joint (A, B), and shorter arrows indicate the abscess. The abscess clearly extends into the medullary region of the bone at the fracture site (e.g., vertical arrows in B and C). The scan was obtained 13 days after the fracture.

**FIGURE 3:**
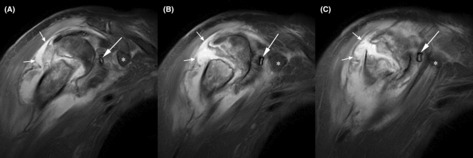
T2 MR images in axial view (A is most posterior, B is just anterior to A, and C is most anterior). Asterisks (*) indicate the coracoid process and the long arrows indicate the Jackson–Pratt drain and the short arrows indicate the abscess. The scan was obtained 13 days after the fracture.

Thirty‐six hours later, grossly purulent fluid in the drain prompted magnetic resonance (MR) scanning with and without intravenous contrast, which revealed a large collection of fluid around and within the fracture site, consistent with osteomyelitis with abscess (Figures 2 and 3). An MRI scan was not obtained earlier, which in retrospect was unfortunate because this delayed definitive control of the source of the infection (discussed further below as a shortcoming in the work‐up of our patient's case). Open and arthroscopic surgical debridement was then done and revealed 30 mL of pus around and within the fracture site. A small tear in the glenohumeral capsule explained how purulent fluid entered the glenohumeral joint. Using a deltopectoral approach, the anterior aspect of the fracture was elevated to allow for debridement of the pus in the medullary region and for the insertion of a hand‐made dowel‐like structure (approximately 7 cm x 0.9 cm) consisting of polymethyl methacrylate (PMMA) cement. This was loaded with tobramycin powder (1.2 g) and vancomycin powder (1.0 g). Antibiotic‐containing PMMA beads (~8 mm diameter) were also placed around the fracture.

**FIGURE 4:**
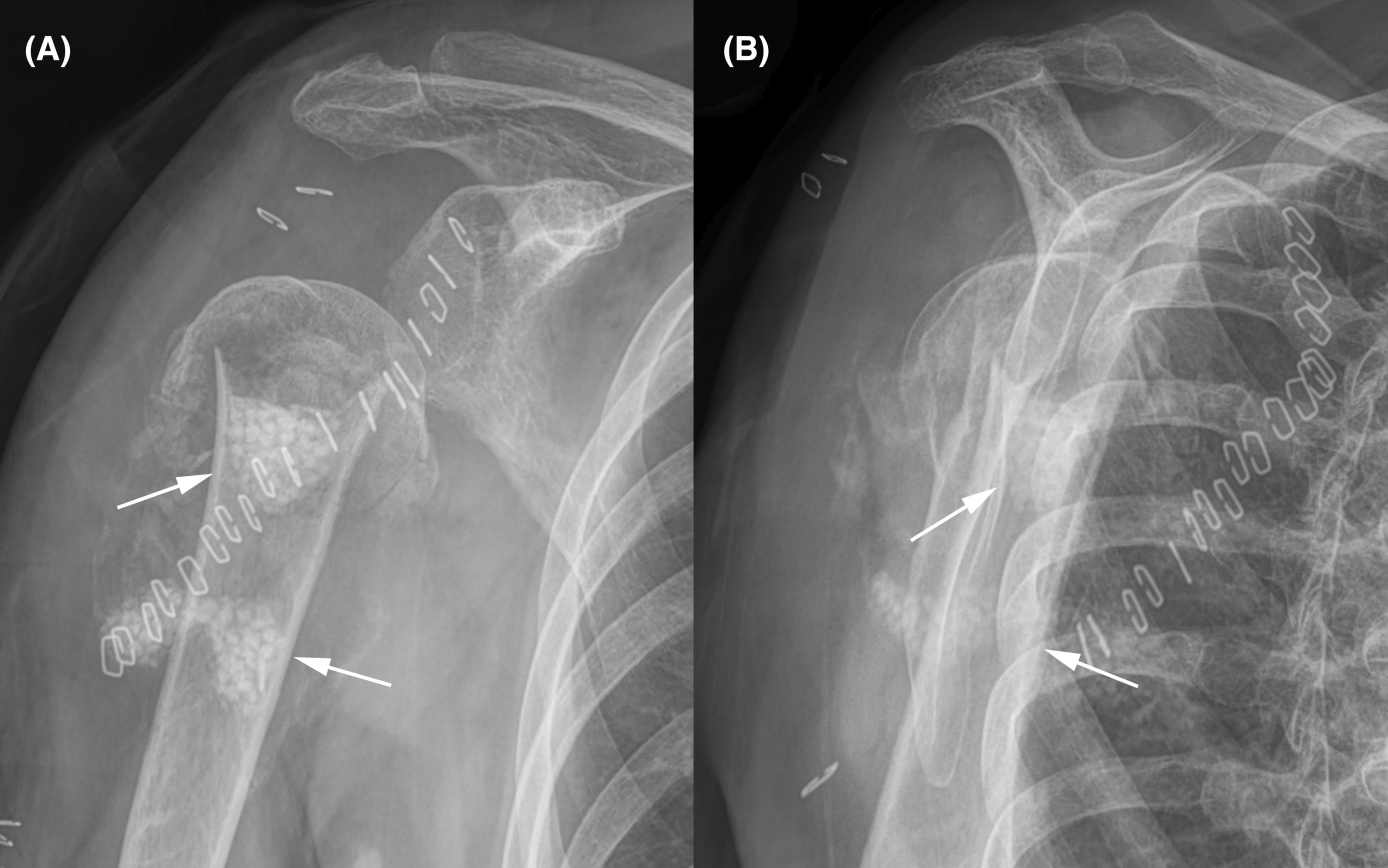
Anterior–posterior (A) and lateral (B) radiographs obtained at 2 weeks after the final debridement surgery (skin staples can be seen). The intramedullary calcium sulfate beads are indicated by the arrows. Note some of the beads subsided into the proximal diaphyseal medullary canal.

The PMMA beads were removed several days later and were replaced with 30 mL of ~3 mm diameter absorbable calcium sulfate beads containing vancomycin and tobramycin (Stimulan®, Biocomposites Ltd., Keele, United Kingdom). To maintain placement of the beads at the fracture site, the medullary canal was occluded with Gelfoam**®** (sterile absorbable gelatin sponge; Pfizer Inc., USA). About 75% of the calcium sulfate beads were then manually packed into the humerus fracture site. The remaining beads were packed around the fracture site (Figure 4). Although stabilization of the fracture with open reduction and internal fixation (ORIF) can help eradicate a bone infection,^17^ we used an alternative method because concerns for bacterial colonization on the hardware and fixation failure due to the poor‐quality bone. The alternative method utilized holes drilled in the anterior, anteromedial, and anterolateral humeral neck and shaft, and figure‐of‐eight (tension band) suturing using No. 2 antibiotic‐containing resorbable monofilament polydioxanone suture,^18^ which reduces the chance of bacterial colonization.^19^, ^20^

**FIGURE 5:**
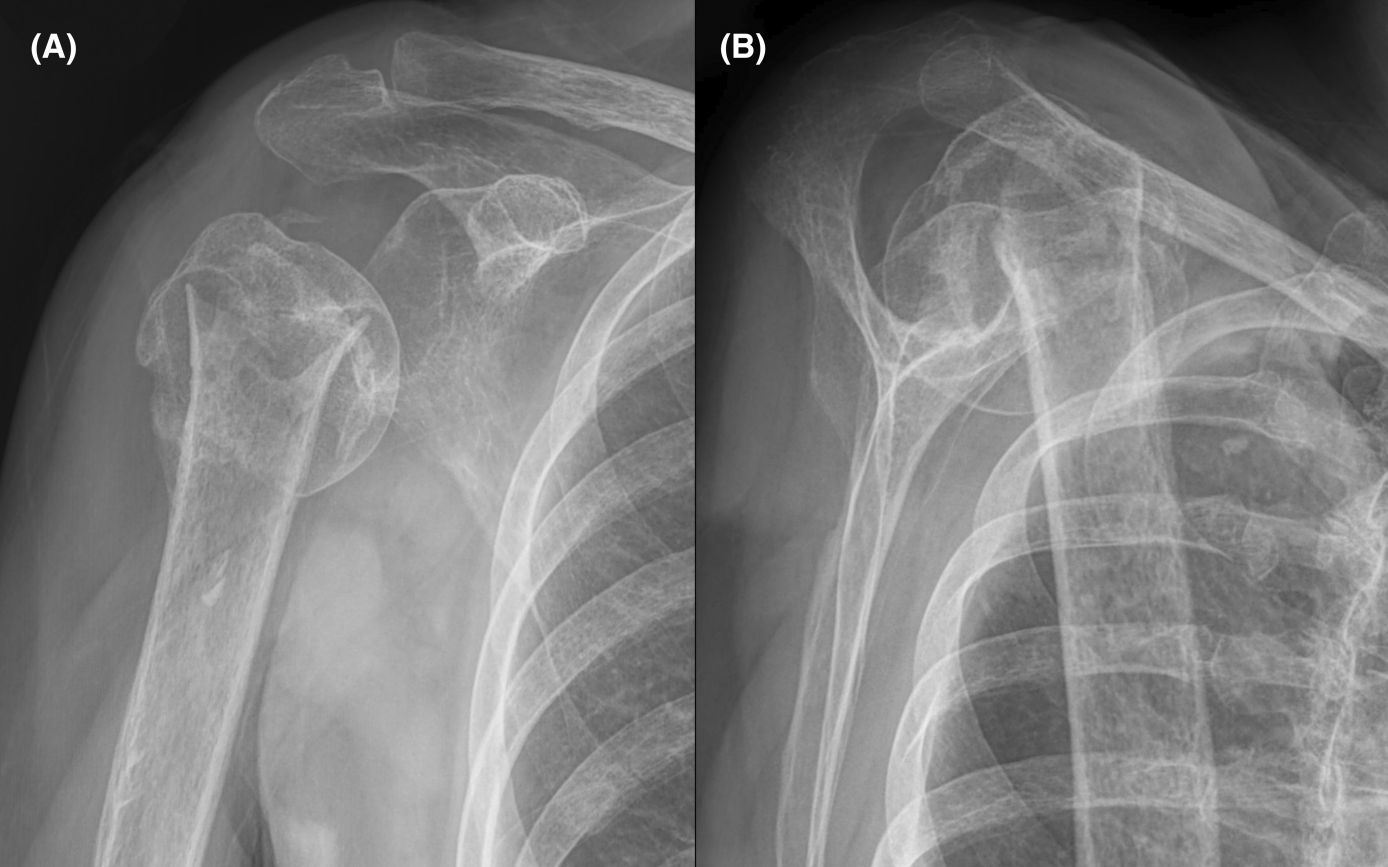
Anterior–posterior (A) and lateral (B) radiographs obtained at 4 months after the final debridement surgery. The fracture is clearly a displaced atrophic nonunion and a pseudarthrosis has formed.

Treatment of the MSSA grown in tissue cultures included IV cefazolin that was given for 6 weeks after the final debridement surgery. The fracture did not heal, the nonunion became painful, and radiographs obtained 4 months later showed a pseudoarthrosis (Figure 5). On February 2, 2021, open surgery was performed to obtain multiple tissue specimens from the nonunion site for cultures in order to rule‐out infection in anticipation for reconstructive surgery.^21^ There was no growth of these multiple cultures at 14 days of incubation.

The patient then decided to seek future care at a local university medical center, which was prompted by her daughter who was employed at that institution. The surgeon at that facility performed a reverse total shoulder arthroplasty (RTSA) on May 19, 2021. At final follow‐up at 24 months after the RTSA (28 months after the final debridement surgery) there was no evidence of infection and the patient was very satisfied with her shoulder function and with her final outcome.

## 3 DISCUSSION

While osteomyelitis can occur in approximately 12%–20% of open fractures in adults,^22^, ^23^ the occurrence of osteomyelitis at the site of a closed fracture is very rare. Our literature review of PubMed and Google Scholar revealed only seven cases of adults who had closed humerus fractures that were complicated with what was reported as “acute osteomyelitis”^3^, ^5^, ^6^, ^7^, ^8^ (Table 1). Of these seven cases, two were of the humeral diaphysis^3^, ^8^ and the other five occurred in the region of the humeral surgical neck. One case isolated penicillin‐resistant *Staphylococcus aureus* from the infected fracture site and was similar to our case (described further below).^7^ Another case isolated methicillin‐resistant *Staphylococcus aureus* (MRSA) with concurrent *Salmonella enteriditis*,^5^ and the remaining four cases reported other causative organisms, including MSSA,^3^ Group B *Streptococcus* (Case 2 from^7^), *Bacteroides* sp. (Case 5 from^7^), and *Escherichia coli*.^6^ *S. aureus* is the most common cause of osteomyelitis in adults.^1^, ^2^, ^24^, ^25^

In our case and in some of these prior cases, it is possible that the osteomyelitis was subacute or chronic, resulting in weakening of the bone. In this theoretical context, these fractures would be considered pathological.^26^, ^27^ In other words, if an intraosseous abscess was already present, then the acute fracture enabled its extraosseous dissemination. This seems unlikely in our patient because of the lack of (1) prior right upper arm or shoulder pain or swelling, (2) local or constitutional symptoms such as warmth, achiness, fevers, chills or sweats, (3) a known locus of chronic infection,^28^ and (4) current or prior laboratory values and vital signs that could raise concern for infection. Additionally, within the 6 months prior her index right humerus fracture, our patient had MR scan of her contralateral (left) shoulder in order to evaluate for a rotator cuff tear after a fall. This suggests that MR scanning was readily available for evaluation of her right shoulder had there been significant symptoms in that region. Definitive proof that our patient's osteomyelitis was acute would require CT or MR scanning within days or a few weeks of the fracture and/or histological examination of the material from the acute fracture site or from the nascent infection site.^29^ Neither of these was done in our patient's case.

The prior reported case that resembles our patient was a 58‐year‐old female that also: (1) was an alcoholic, (2) had a closed proximal humerus fracture from a ground‐level fall, and (3) had *S. aureus* (likely MSSA) as the causative organism (Case 1 in^7^). Treatment then (early 1970s) did not include the use of absorbable calcium sulfate beads, and nonabsorbable tobramycin‐impregnated PMMA beads were just being introduced. Although that patient's infection was eradicated, humeral head resection was the treatment for the painful nonunion.

Although our patient's nonunion may have resulted from compromised blood flow at the time of fracture,^30^, ^31^ the displacement of fracture that was done to insert a PMMA dowel and to place calcium sulfate beads likely further perturbed blood flow. This is important because these beads require adequate blood supply to enable bone healing.^32^, ^33^ Surgical perturbation of the fracture site could have been avoided if the metaphyseal region had been debrided through a drill hole made near the supraspinatus tendon insertion.^34^ Drilling in this location (as when employing intramedullary nailing for proximal two‐part humerus fractures) does not compromise humeral head blood flow.^35^ Although this is an established method for debriding the medullary canal of the humerus and other long bones,^16^, ^36^ we felt that it would not have allowed for adequate debridement in our case (Figure 2).

## 4 CONCLUSION

We describe a rare case of an infection at the site of a closed acute proximal humerus fracture in an adult with chronic alcoholism. Whether or not this infection was acute, subacute, or chronic, it is rare to have a deep infection at the site of a closed fracture in an adult. When faced with similar circumstances of unexplained leukocytosis, malaise, fever, and increasing pain at a fracture site, and/or positive blood cultures in an immunocompromised patient, healthcare providers should consider immediate MR or CT scanning to help determine if an infection is present at the fracture site.

## AUTHOR CONTRIBUTIONS

**John G. Skedros:** Conceptualization; data curation; investigation; supervision; writing – original draft; writing – review and editing. **Tyler R. Smith:** Data curation; writing – original draft; writing – review and editing. **John T. Cronin:** Data curation; writing – original draft; writing – review and editing.

## FUNDING INFORMATION

No funding was obtained for this case report.

## CONFLICT OF INTEREST STATEMENT

The authors have no conflict of interest to declare.

### ETHICAL APPROVAL

Each author certifies that his institution has approved the reporting of this case, that all investigations were conducted in conformity with ethical principles of research, and that informed consent for participation in the study was obtained.

### CONSENT

Written informed consent was obtained from the patient to publish this report in accordance with the journal's patient consent policy.

## Data Availability

The data that support the findings of this study are available from the corresponding author upon reasonable request.

## References

[1] Grigorian A , Schubl S , Scolaro J , et al. No increased risk of acute osteomyelitis associated with closed or open long bone shaft fracture. J Clin Orthop Trauma. 2019;10(Suppl 1):S133‐S138. 31700209 10.1016/j.jcot.2019.04.003 PMC6823910

[2] Kocutar T , Snoj Z , Salapura V . Complicated acute haematogenous osteomyelitis with fatal outcome following a closed clavicle fracture‐a case report and literature review. BJR Case Rep. 2016;2(2):20150389. 30363605 10.1259/bjrcr.20150389 PMC6180849

[3] Gellman YN , el‐Haj M , Khoury A , Weil YA . Closed humeral fracture complicated with acute hematogenous osteomyelitis: a case report. J Orthop Case Rep. 2018;8(2):61‐64. 10.13107/jocr.2250-0685.1052 PMC6114204 30167416

[4] Kim C , Tufescu TV . Infection in closed fractures: a case report and literature review. JBJS Case Connect. 2012;2(3):e44. 29252542 10.2106/JBJS.CC.L.00008

[5] Aluisio FV , Scully SP . Acute hematogenous osteomyelitis of a closed fracture with chronic superinfection. Clin Orthop Relat Res. 1996;325:239‐244. 10.1097/00003086-199604000-00029 8998882

[6] Martίnez AR , Duca A , Rubio EM , Herreros MLV , Feito CR . Osteomielitis por Eschericia coli sobre fractura cerrada de húmero [in Spanish]. Anales de Medicina Interna (Madrid). 2006;23(12):588‐590. 10.4321/s0212-71992006001200008 17371148

[7] Watson FM , Whitesides TE Jr . Acute hematogenous osteomyelitis complicating closed fractures. Clin Orthop Relat Res. 1976;117:296‐302. 1277679

[8] Baruah RK , Kumar S , Haque R . Acute osteomyelitis in closed fracture of adult humerus‐ case report and review of the literature. J Dental Med Sciences. 2016;15(3):31‐34.

[9] Cozen L . Four unusual cases of osteomyelitis in adults. West J Surg Obstet Gynecol. 1958;66(1):36‐39. 13507363

[10] Weidle PA , Brankamp J , Dedy N , Haenisch C , Windolf J , Jonas M . Complication of a closed Colles‐fracture: necrotising fasciitis with lethal outcome. A case report. Arch Orthop Trauma Surg. 2009;129(1):75‐78. 18931852 10.1007/s00402-008-0748-x

[11] Baskaran S , Nahulan T , Kumar AS . Close fracture complicated by acute haematogenous osteomyelitis. Med J Malaysia. 2004. 59(Suppl F):72‐74. 15941170

[12] Wald ER . Risk factors for osteomyelitis. Am J Med. 1985;78(6B):206‐212. 3893117 10.1016/0002-9343(85)90386-9

[13] Thein R , Tenenbaum S , Chechick O , Leshem E , Chechik A , Liberman B . Delay in diagnosis of femoral hematogenous osteomyelitis in adults: an elusive disease with poor outcome. Isr Med Assoc J. 2013;15(2):85‐88. 23516768

[14] Berg KM , Kunins HV , Jackson JL , et al. Association between alcohol consumption and both osteoporotic fracture and bone density. Am J Med. 2008;121(5):406‐418. 18456037 10.1016/j.amjmed.2007.12.012 PMC2692368

[15] Prodinger PM , Pilge H , Banke IJ , et al. Acute osteomyelitis of the humerus mimicking malignancy: Streptococcus pneumoniae as exceptional pathogen in an immunocompetent adult. BMC Infect Dis. 2013;13:266. 23738890 10.1186/1471-2334-13-266 PMC3679722

[16] Wu H , Yu S , Fu J , et al. Investigating clinical characteristics and prognostic factors in patients with chronic osteomyelitis of humerus. Burns Trauma. 2019;7:34. 31844634 10.1186/s41038-019-0173-0 PMC6894245

[17] Foster AL , Moriarty TF , Zalavras C , et al. The influence of biomechanical stability on bone healing and fracture‐related infection: the legacy of Stephan Perren. Injury. 2021;52(1):43‐52. 32620328 10.1016/j.injury.2020.06.044

[18] Dimakopoulos P , Panagopoulos A , Kasimatis G . Transosseous suture fixation of proximal humeral fractures. J Bone Joint Surg Am. 2007;89(8):1700‐1709. 17671007 10.2106/JBJS.F.00765

[19] Fowler JR , Perkins TA , Buttaro BA , Truant AL . Bacteria adhere less to barbed monofilament than braided sutures in a contaminated wound model. Clin Orthop Relat Res. 2013;471(2):665‐671. 23001503 10.1007/s11999-012-2593-z PMC3549181

[20] Chu CC , Williams DF . Effects of physical configuration and chemical structure of suture materials on bacterial adhesion. A possible link to wound infection. Am J Surg. 1984;147(2):197‐204. 6364858 10.1016/0002-9610(84)90088-6

[21] Tashjian RZ , Granger EK , Zhang Y . Utility of prerevision tissue biopsy sample to predict revision shoulder arthroplasty culture results in at‐risk patients. J Shoulder Elbow Surg. 2017;26(2):197‐203. 27727058 10.1016/j.jse.2016.07.019

[22] Momodu II , Savaliya V . *Osteomyelitis*. StatPearls [Internet]. https://www.ncbi.nlm.nih.gov/books/NBK532250/ 2021.

[23] Fernandes MDC , Peres LR , de Queiroz AC Jr , et al. Open fractures and the incidence of infection in the surgical debridement 6 hours after trauma. Acta Ortop Bras. 2015;23(1):38‐42. 26327794 10.1590/1413-78522015230100932 PMC4544519

[24] Hadjipavlou AG , Mader JT , Necessary JT , Muffoletto AJ . Hematogenous pyogenic spinal infections and their surgical management. Spine (Phila Pa 1976). 2000;25(13):1668‐1679. 10870142 10.1097/00007632-200007010-00010

[25] Kumar A , Sandoe J , Kumar N . Three cases of vertebral osteomyelitis caused by Streptococcus dysgalactiae subsp. equisimilis. J Med Microbiol. 2005;54(Pt 11):1103‐1105. 16192443 10.1099/jmm.0.46061-0

[26] Krebs NM , Krebs RC , Yaish AM . Femoral osteomyelitis presenting as a pathologic fracture in a 53 year old male: a rare case report. J Orthop Case Rep. 2017;7(6):85‐88. 29600219 10.13107/jocr.2250-0685.962 PMC5868893

[27] Gelfand MS , Cleveland KO , Goswami R , Heck RK . Pathological fracture in acute osteomyelitis of long bones secondary to community‐acquired methicillin‐resistant Staphylococcus aureus: two cases and review of the literature. Am J Med Sci. 2006;332(6):357‐360. 17170628 10.1097/00000441-200612000-00010

[28] Walmsley BH . Pathological fracture in acute osteomyelitis in an adult. J R Soc Med. 1983;76(1):77‐78. 6827504 10.1177/014107688307600118 PMC1438559

[29] Tiemann A , Hofmann GO , Krukemeyer MG , Krenn V , Langwald G . Histopathological Osteomyelitis Evaluation Score (HOES) – an Innovative Approach to Histopathological Diagnostics and Scoring of Osteomyelitis. GMS Interdiscip Plast Reconstr Surg DGPW. 2014;3:Doc08. 26504719 10.3205/iprs000049 PMC4582515

[30] Gerber C , Schneeberger AG , Vinh TS . The arterial vascularization of the humeral head. An anatomical study. J Bone Joint Surg Am. 1990;72(10):1486‐1494. 2254356

[31] Hertel R , Hempfing A , Stiehler M , Leunig M . Predictors of humeral head ischemia after intracapsular fracture of the proximal humerus. J Shoulder Elbow Surg. 2004;13(4):427‐433. 15220884 10.1016/j.jse.2004.01.034

[32] Masrouha KZ , Raad ME , Saghieh SS . A novel treatment approach to infected nonunion of long bones without systemic antibiotics. Strategies Trauma Limb Reconstr. 2018;13(1):13‐18. 29380256 10.1007/s11751-018-0303-4 PMC5862710

[33] Strocchi R , Orsini G , Iezzi G , et al. Bone regeneration with calcium sulfate: evidence for increased angiogenesis in rabbits. J Oral Implantol. 2002;28(6):273‐278. 12498535 10.1563/1548-1336(2002)028<0273:BRWCSE>2.3.CO;2

[34] Johnston PS , Hatzidakis AM , Tagouri YM , Curran‐Everett D , Sears BW . Anatomic evaluation of radiographic landmarks for accurate straight antegrade intramedullary nail placement in the humerus. JSES Int. 2020;4(4):745‐752. 33345210 10.1016/j.jseint.2020.06.004 PMC7738442

[35] Sears BW , Johnston PS , Garrigues GE , Boileau P , Hatzidakis AM . Intramedullary nailing of the proximal humerus – not just for 2‐part fractures. Annals of Joint. 2020;5:1‐8.

[36] Kanakaris N , Gudipati S , Tosounidis T , Harwood P , Britten S , Giannoudis PV . The treatment of intramedullary osteomyelitis of the femur and tibia using the reamer‐irrigator‐aspirator system and antibiotic cement rods. Bone Joint J. 2014;96‐B(6):783‐788. 10.1302/0301-620X.96B6.32244 24891579

